# UWHVF: A Real-World, Open Source Dataset of Perimetry Tests From the Humphrey Field Analyzer at the University of Washington

**DOI:** 10.1167/tvst.11.1.1

**Published:** 2022-01-03

**Authors:** Giovanni Montesano, Andrew Chen, Randy Lu, Cecilia S. Lee, Aaron Y. Lee

**Affiliations:** 1 City, University of London, Optometry and Visual Sciences, London, UK; 2 NIHR Biomedical Research Centre, Moorfields Eye Hospital NHS Foundation Trust and UCL Institute of Ophthalmology, London, UK; 3 Department of Ophthalmology, University of Washington, Seattle, Washington, USA

**Keywords:** humphrey visual field, glaucoma, dataset, open source, HVF

## Abstract

**Purpose:**

This article describes the Humphrey field analyzer (HFA) dataset from the Department of Ophthalmology at the University of Washington.

**Methods:**

Pointwise sensitivities were extracted from HFA 24-2, stimulus III visual fields (VF). Total deviation (TD), mean TD (MTD), pattern deviation, and pattern standard deviation (PSD) were calculated. Progression analysis was performed with simple linear regression on global, regional, and pointwise values for VF series with greater than four tests spanning at least four months. VF data were extracted independently of clinical information except for patient age, gender, and laterality

**Results:**

This dataset includes 28,943 VFs from 7248 eyes of 3871 patients. Progression was calculated for 2985 eyes from 1579 patients. Median [interquartile range] age was 64 years [54, 73], and follow-up was 2.49 years [1.11, 5.03]. Baseline MTD was −4.51 dB [−8.01, −2.65], and baseline PSD was 2.41 dB [1.7, 5.34].

**Conclusion:**

MTD was found to decrease by −0.10 dB/yr [−0.40, 0.11] in eyes for which progression analysis was able to be performed. VFs with deep localized defects, PSD > 12 dB and MTD −15 dB to −25 dB, were plotted, visually inspected, and found to be consistent with neurologic or glaucomatous VFs from patients. For a small number of tests, extracted sensitivity values were compared to corresponding printouts and confirmed to match.

**Translational Relevance:**

This open access pointwise VF dataset serves as a source of raw data for investigation such as VF behavior, clinical comparisons to trials, and development of new machine learning algorithms.

## Introduction

Glaucoma is an optic neuropathy defined by characteristic change of the optic nerves with corresponding visual field (VF) deficits. VF testing with standard automated perimetry plays an integral role in the assessment and management of patients with glaucoma by allowing providers to track patients’ visual function and estimate future decline.

More recently, there has been a growing interest in applying artificial intelligence (AI) to the arena of VF analysis to forecast future fields,^1^ identify common glaucomatous field defects,^2^ or detect the presence of glaucomatous progression,^3^ as a few examples. As with other applications of AI, meaningful data of sufficient scale is required to adequately train the AI for its intended purpose. Significant work is required to prepare these datasets for analysis, and the limited access to this data presents a barrier to researchers interested in studying VFs. The ability to have access to an open dataset could significantly accelerate VF research.^4^

This article describes the open-source VF dataset from the University of Washington and the steps involved in processing and annotating the raw data. This repository is published and available at https://github.com/uw-biomedical-ml/uwhvf.

## Methods

### Data Extraction

Standard automated perimetry tests from all patients performed on the Humphrey field analyzer (HFA) II (Carl Zeiss Meditec, Inc. Dublin, CA, USA) at the University of Washington were extracted under an Institutional Review Board–approved protocol and then all protected health information was destroyed to create a deidentified dataset for public release. All VF testing dates were converted to floating point years of age by calculating the days of life from birth to the date of the VF testing and then dividing by 365.25. All ages above 90 were changed to be 90 to be in accordance with HIPAA Safe Harbor guidelines for deidentification. Floating point estimations of VF sensitivities at each testing location were extracted from the binary header data for the VF file by decoding the hexadecimal values as little endian integers. Duplicated VFs were identified by finding VF instances with the exact same sensitivities at each location with the same age and same eye. Duplicated series were merged together and the data was formatted into JSON for public release. All tests were 24-2 white-on-white Goldmann stimulus size III examinations, performed with either a Swedish Interactive Thresholding Algorithm (Standard or Fast) or a full-threshold strategy.

### Derived Metrics

Total deviation (TD) values were calculated using normative values from an HFA. These values were obtained by running mock tests on the device where no response was provided and inputting different ages, by decade. The TD maps in these mock tests therefore reported the (Normative value) for each location at each age. TD values were calculated for all 52 locations in the 24-2 HVF, excluding the two blind spots at (X = 15; Y = ±3) degrees from fixation (for a right eye). The pattern deviation (PD) was calculated by subtracting the seventh highest (less negative) TD value from all the other TD values. The seventh highest value is used as a robust estimate of the general height (GH) of the field, which is then used to account for generalized depression of the sensitivity because of, for example, optical media opacity. Our calculation reflects the definition provided by the Imaging and Perimetry Society.^5^^,^^6^

Global indexes were also calculated. The usual HFA mean deviation (MD) makes use of a weighting system based on location-specific variability estimates,^5^ which cannot be extracted from the HFA device. Therefore we calculated the mean total deviation (MTD) instead, which is simply the arithmetic average of the TD values. Its interpretation is essentially equivalent to MD.^7^^,^^8^ Of note, this is identical to the definition of mean defect on Octopus perimeters.^5^ Similarly, pattern standard deviation (PSD) was also calculated as the standard deviation of the TD values, without any correction factors.^5^ The mean sensitivity (MS) was calculated as the average of the 52 sensitivity values (excluding the two blind-spot locations) in the 24-2 VF. Average localized perimetric defect was also quantified for each VF cluster by calculating clusterwise MTD and MS, as described by Garway-Heath et al.^9^

### Quantification of Progression

Global, clusterwise, and pointwise progressions of VF defect over time were quantified using simple linear regression on TD values or their cluster or global average. Progression was only calculated on a subset of eyes with a minimum of four tests spanning at least four months. This selection was made to reduce large fluctuations in the progression slopes, particularly when only a few tests were concentrated over a short period of time.

## Data Summary

**Figure 1.**
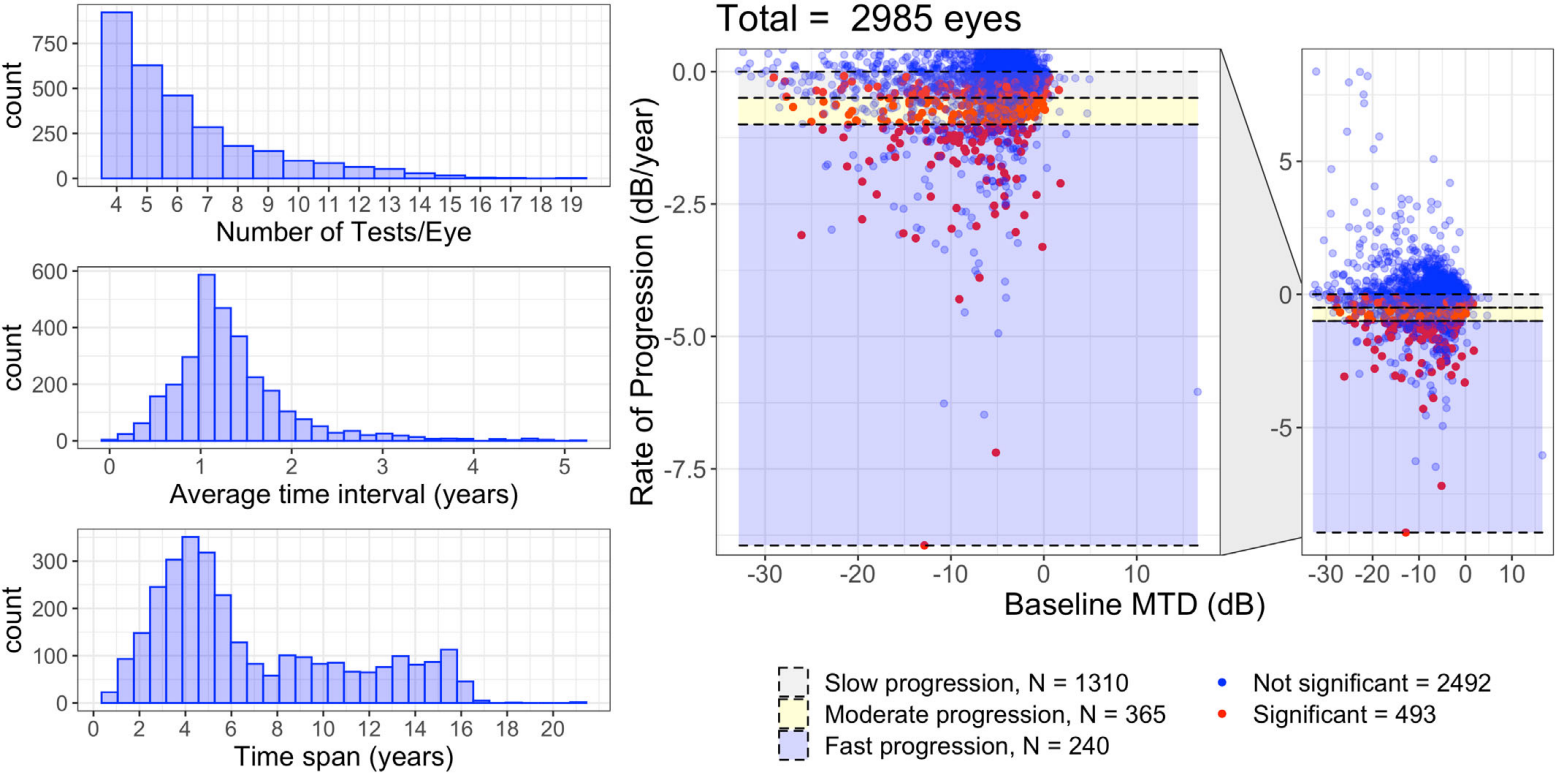
Descriptive statistics for the 2985 eyes for which progression could be calculated. Significant: *P* < 0.05 and negative slope. Rate of progression is for the MTD. For classification of the rate of progression, see text.

The database includes 28943 VFs from 7248 eyes of 3871 patients. Descriptive statistics for the whole sample are reported in Table 1. Progression was calculated for 2985 eyes from 1579 patients. Figure 1 reports additional descriptive statistics for the eyes that progressed and represents the distribution of MTD slopes with respect to their baseline value.

**Table 1.** Descriptive Statistics of the Sample


Baseline age (y), median [interquartile range]	64 [54, 73]
Gender	
Female	1608 (41%)
Male	1390 (35%)
Unspecified	933 (24%)
Follow-up length (y), median [interquartile range]	2.49 [1.11, 5.03]
Number of tests (per eye), median [interquartile range]	3 [2, 5]
Average intertest interval (y), median [interquartile range]	1.13 [0.84, 1.62]
Baseline PSD (dB), median [interquartile range]	2.41 [1.70, 5.34]
Baseline MTD (dB), median [interquartile range]	
Global	−4.51 [−8.01, −2.65]
Cluster 1 (Superior peripheral)	−4.60 [−9.01, −2.22]
Cluster 2 (Superior paracentral)	−4.51 [−8.02, −2.59]
Cluster 3 (Central nasal)	−3.56 [−6.36, −2.12]
Cluster 4 (Inferior paracentral)	−4.10 [−6.92, −2.56]
Cluster 5 (Inferior peripheral)	−4.45 [−8.01, −2.54]
Cluster 6 (Temporal)	−4.08 [−7.23, −2.17]

Clusters are defined as in Garway-Heath et al.^9^ Average intertest interval was only computed for eyes with more than one test (N = 7398).

Progression was classified as slow (0 dB/year > slope ≥ −0.5 dB/year), moderate (−0.5 dB/year > slope ≥ −1 dB/year) and fast (slope < −1 dB/year).

This dataset is open sourced under the three-clause Berkeley Source Distribution license. In addition, as recommended by Gebru et al.,^10^ we have provided a structured datasheet in Supplementary Materials.

### Raw Data

The raw dataset is provided in two alternative formats:

1) Structured JSON file: this file contains sensitivity values, TD values, age, laterality (left or right eye), and gender when specified. Sensitivity and TD values are stored both in long format (as a vector) and provided as an 8 × 9 matrix. The latter is meant to preserve the original spatial organization of the data, which is particularly useful in spatial-aware processing often used in machine learning. All VF data are stored as a right eye in that the left eye VFs are flipped to have the same layout as the right eye. Empty matrix cells are filled with a fixed value (100). A validated JSON Schema is provided in the repository for full description of the data.
2) Long format table: this is a comma-separated value (CSV) file, where each row contains data from one test. Sensitivity and TD values are reported in long format, in the same order as in the JSON file. This file contains additional information, such as the GH, the PD values, the time from baseline (in years) and clusterwise and global metrics. The VF coordinates for each location (right eye format) are provided in a separate CSV file.

### Progression Data

Additional progression and baseline data are reported separately for each eye. Global progression is reported in a CSV file. Clusterwise and pointwise intercepts, progression slopes and *P* values are reported in separate tables, where each row corresponds to an individual eye and each column to an individual location/cluster. Locations are ordered as previously described. Clusters follow those defined by Garway-Heath et al.^9^ In short, the clusters are labeled as cluster 1 (superior peripheral), cluster 2 (superior paracentral), cluster 3 (central nasal), cluster 4 (inferior paracentral), cluster 5 (inferior peripheral), and cluster 6 (temporal). For consistency, eyes for which the calculation of progression was not possible (see selection criteria described above) are reported in the table, but the corresponding cells for progression metrics are left empty.

## Technical Validation

**Figure 2.**
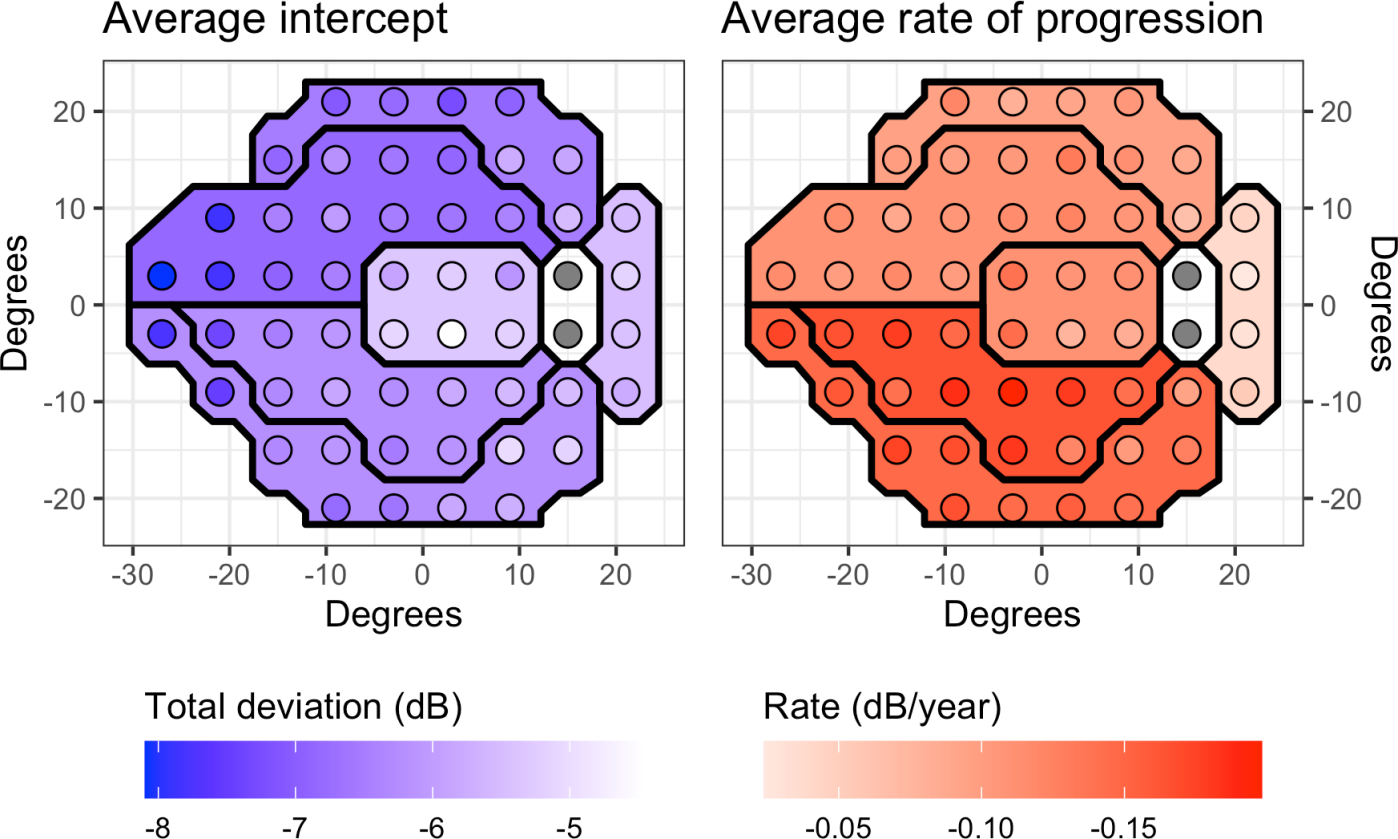
Average baseline damage and rate of progression (slope) for total deviation by location and cluster.

Additional descriptive statistics for progression slopes and intercepts (global and by cluster) are reported in Table 2. Regional differences in baseline VF defect and the progression rate are shown in Figure 2. The average difference in rate of progression between MS and MTD was −0.06 dB/year, which is in excellent agreement with the average normal VF ageing reported by Spry and Johnson (−0.064 dB/year).^11^

**Table 2.** Median [Interquartile Range] Statistics for Global and Clusterwise Parameters From Linear Regression

	MS, Intercept (dB)	MS, Slope (dB/year)	MTD, Intercept (dB)	MTD, Slope (dB/year)
Global	27.64 [23.91, 29.55]	−0.16 [−0.45, 0.05]	−4.28 [−7.79, −2.48]	−0.10 [−0.40, 0.11]
Cluster 1	25.29 [20.89, 27.73]	−0.16 [−0.53, 0.14]	−4.19 [−8.72, −2.04]	−0.08 [−0.46, 0.21]
Cluster 2	27.56 [23.91, 29.48]	−0.13 [−0.45, 0.09]	−4.33 [−7.82, −2.48]	−0.07 [−0.39, 0.15]
Cluster 3	30.53 [27.73, 32.07]	−0.13 [−0.44, 0.08]	−3.47 [−6.21, −2.05]	−0.07 [−0.38, 0.14]
Cluster 4	29.34 [26.51, 30.93]	−0.15 [−0.43, 0.05]	−3.91 [−6.63, −2.42]	−0.10 [−0.38, 0.10]
Cluster 5	27.38 [24.10, 29.31]	−0.16 [−0.48, 0.06]	−4.12 [−7.33, −2.31]	−0.11 [−0.43, 0.11]
Cluster 6	27.54 [24.75, 29.34]	−0.12 [−0.45, 0.14]	−3.84 [−6.52, −2.12]	−0.06 [−0.39, 0.20]

Clusters are defined as in Garway-Heath et al.^9^

**Figure 3.**
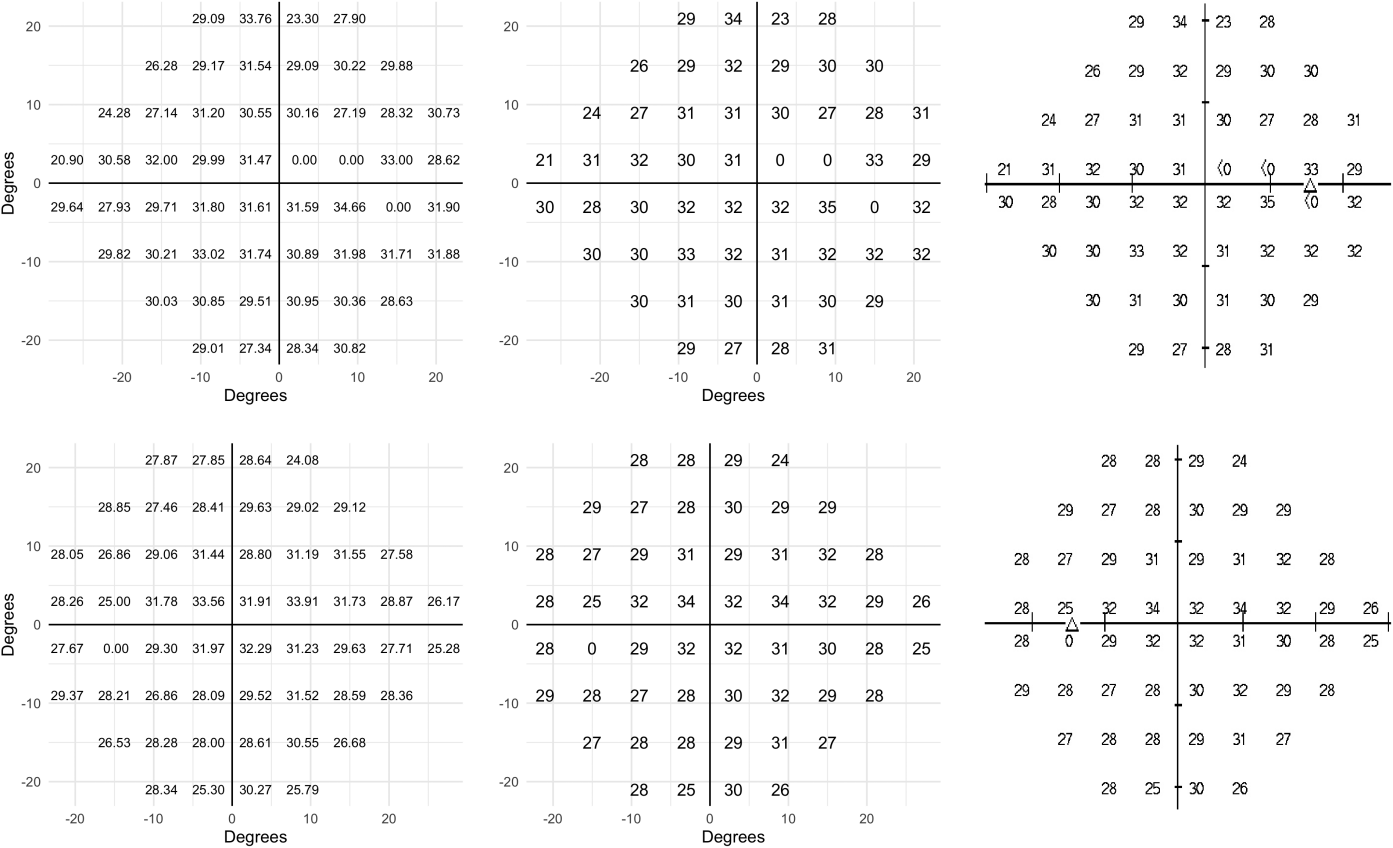
Two examples of visual field sensitivity from the dataset (*left panels*), their rounded value (*middle panels*), and the corresponding sensitivity plots on the original printouts (*right panels*).

A selection of 315 VFs in the dataset were plotted and visually inspected by two experts. We targeted VFs with a high likelihood of localized deep defects by selecting examples with a PSD > 12 dB and a MTD between −15 dB and −25 dB. The two experts evaluated the plausibility of the plotted examples, looking for typical glaucomatous or neurological patterns. All plots were found to be consistent with bona fide real VF test results. Because our data were deidentified, at the time of extraction we were not able to link them back to the original printouts. However, for two examples, the original HFA printouts were extracted manually without deidentification for validation purposes. These are reported in Figure 3.

## Discussion

We present an open-sourced, observational VF dataset curated from a single academic institution with progression analysis performed on series with at least four VF tests over at least four months. The raw sensitivities and progression of pointwise, clusterwise and global values are included in the raw data files. To our knowledge, this is the first open access VF dataset of this magnitude to be made available for research.

Rates of change in this dataset are in line with those presented previously. Spry and Johnson^11^ reported a progression rate of 0.64 dB per decade in normal eyes, which is in line with the difference we found between the MS and MTD progression rate. They also reported that the rate may be affected by age, eccentricity of the test location, and the hemifield.^11^ In this dataset, the most damage at baseline occurred in the superior hemifield (Fig. 2), in agreement with previous reports.^12^^,^^13^ However, in our dataset, the inferior field was the fastest progressing region. Other studies in glaucoma patients report a wide range for mean deviation rate of progression from −0.05 to −0.57 dB/year.^14^^–^^20^ The variability of MD rates could be influenced by factors such as different baseline glaucoma severity, surgical interventions during the follow-up period, and different follow-up times. Although MD is not available in this dataset because of proprietary location-specific variability estimates, the surrogate mean total deviation has been shown to be equvalent in interpretation.^7^^,^^8^

Pointwise sensitivities were extracted from the binary header data in the VF file for this dataset. Pointwise sensitivities can also be obtained by exporting the data from Zeiss Forum or, more recently, by extracting values from the DICOM file or images of the VF report with third-party software.^21^^,^^22^ Zeiss Forum was not available at our institution at the time of data extraction. Duplicate tests from the extraction process used for this dataset had to be identified and removed. These duplicate tests likely occurred due to a combination of correction of user input errors, relocations of devices across different sites, or transitions between data servers across the years.

This open access VF dataset is the first of its size to be published and aims to lower the barrier to entry for the scientific community.^4^ We provide both summary statistics and technical validation of our dataset. An open-access VF dataset would have a number of applications. It could be used to explore localized rates of change and interactions of neighboring VF locations. It can serve as a clinically-derived point of comparison to other VF datasets from other studies or for future clinical trials. It could also be used to evaluate the effects of different criteria for progression analysis. The size of this dataset opens avenues for possible machine learning applications.

Limitations to this dataset exist. First, the VF data were extracted independently of clinic information other than patient age, gender, and laterality. Some clinical information was not included, such as initiation and timing of treatment and surgical interventions. The dataset represents all patients undergoing VF testing at an academic institution with or without glaucoma diagnosis, and may not reflect the general patient population. Further work will have to be performed to identify, categorize, and rectify relevant health records. Second, reliability indices were not extracted, but the effects of less-reliable tests would be somewhat mitigated by the number of eyes and tests in this dataset. Third, proprietary information in the HFA limited the information that could be extracted. Probability deviation maps were not available, along with the Glaucoma Hemifield Test classification and Statpac analysis such as the Guided Progression Analysis, which are used clinically to help determine glaucoma progression and diagnosis. Likewise, our TD and PD values were not directly extracted from the test but rather calculated from the sensitivity values. However, as explained in the Methods section, the TD maps were derived as deviations from the normative values extracted from the machine and are therefore likely to be accurate. We confirmed this by showing that the difference between the average rate of progression for MS and MTD matched the expected sensitivity decline because of aging alone (−0.06 dB/year).^11^ PD maps were derived according to the Imaging and Perimetry Society standards from TD maps. Therefore, provided that the TD values are correct, they should be reflective of what would be obtained from the actual HFA printouts. It is also worth mentioning that other freely available software, such as the visualFields package for R,^23^ provide independent normative datasets and tools to calculate all these metrics from any dataset, including ours. It should be finally noted that we used simple linear regression to quantify progression. Better and more precise methods exist.^24^^–^^28^ However, the scope of our progression analysis was mainly to provide descriptive statistics of the sample for researchers making use of our database, for example to select specific patients based on their rate of progression. As such, we chose simple linear regression as a straightforward method, easily replicated by other researchers for validation of their results. On the other hand, because we are making our database fully available, researchers in different fields would be able to apply their preferred method for detection of progression for their specific applications.

The transition of health data to an electronic health record format opens the possibility of access to large datasets for research. Large, high-quality datasets have provided the foundation for innovative statistical and computational models but also present a barrier of entry. This VF dataset aims to help establish an open access repository so that the scientific community may use it to accelerate discoveries in this field.

## Supplementary Material

Supplement 1
