# Supplementary material for: UWHVF: A Real-World, Open Source Dataset of Perimetry Tests From the Humphrey Field Analyzer at the University of Washington
Source: Transl Vis Sci Technol. 2022 Jan 3;11(1):1. doi: 10.1167/tvst.11.1.1 (PMC8742531; doi:10.1167/tvst.11.1.1)
Supplement: Supplement 1 [file tvst-11-1-1_s001.pdf]

Supplementary Material: Datasheet for Dataset

Motivation

For what purpose was the dataset created?
Meaningful data of sufficient scale is required to adequately train the AI for its intended purpose, and significant work is required to prepare these datasets. This open access visual field data set curated from a single academic institution is the first of its size to be published. We aim to lower the barrier to entry for the scientific community and increase accessibility for visual field and machine learning researchers.

Who created the dataset (e.g., which team, research group) and on behalf of which entity (e.g., company, institution, organization)?
University of Washington

Who funded the creation of the dataset?
NIH/NEI K23EY029246 (Bethesda, MD), NIH/NIA R01AG060942 (Bethesda, MD), Latham Vision Research Innovation Award (Seattle, WA), and an unrestricted grant from Research to Prevent Blindness (New York, NY).

Composition

What do the instances that comprise the dataset represent (e.g., documents, photos, people, countries)?
Humphrey Visual Field data consisting of perimetry sensitivities

How many instances are there in total (of each type, if appropriate)?
28,943

What data does each instance consist of?
Standard Automated Microperimetry sensitivity value (dB)

Is there a label or target associated with each instance?
No

Is any information missing from individual instances?
No

Are there recommended data splits (e.g., training, development/validation, testing)?
No

Are there any errors, sources of noise, or redundancies in the dataset?
Duplicated HVFs (exact same sensitivities at each location with the same age and same eye) were identified and merged together

Is the dataset self-contained, or does it link to or otherwise rely on external resources (e.g., websites, tweets, other datasets)?
This dataset is self-contained.

Does the dataset contain data that might be considered confidential (e.g., data that is protected by legal privilege or by doctor–patient confidentiality, data that includes the content of individuals' non-public communications)?
The dataset was extracted under an IRB approved protocol and then all protected health information was destroyed to create a deidentified dataset for public release.

Does the dataset relate to people?
Yes

Does the dataset identify any subpopulations (e.g., by age, gender)?
There are no subpopulations defined; however, the data set represents all patients undergoing visual field testing at an academic institution, and may not reflect the general patient population.

Collection Process

How was the data associated with each instance acquired?
24-2 HVF data were collected with standard automated perimetry

What mechanisms or procedures were used to collect the data (e.g., hardware apparatus or sensor, manual human curation, software program, software API)?
All tests were 24-2 white-on-white Goldmann stimulus size III exams, performed with either a Swedish Interactive Thresholding Algorithm (SITA, Standard or Fast) or a Full-threshold strategy.

Who was involved in the data collection process (e.g., students, crowdworkers, contractors) and how were they compensated (e.g., how much were crowdworkers paid)?
Data was collected by ophthalmologic technicians working at the University of Washington as part of routine clinical care.

Over what timeframe was the data collected?
Data was collected between 1998 and 2018

Were any ethical review processes conducted (e.g., by an institutional review board)?
Yes - data was extracted under an IRB approved protocol

Did you collect the data from the individuals in question directly, or obtain it via third parties or other sources (e.g., websites)?
Data was collected from the clinical information systems directly

Were the individuals in question notified about the data collection?
No, waived by IRB

Did the individuals in question consent to the collection and use of their data?
No, waived by IRB

If consent was obtained, were the consenting individuals provided with a mechanism to revoke their consent in the future or for certain uses?
N/A

Has an analysis of the potential impact of the dataset and its use on data subjects (e.g., a data protection impact analysis) been conducted?
No

Preprocessing/cleaning/labeling

Was any preprocessing/cleaning/labeling of the data done (e.g., discretization or bucketing, tokenization, part-of-speech tagging, SIFT feature extraction, removal of instances, processing of missing values)?
In addition to the raw data, some derived metrics listed below were also calculated and included in the dataset:

- Total Deviation (TD) values were calculated using normative values from a Humphrey Field analyzer (HFA) II device
- The Pattern Deviation (PD) was calculated by subtracting the General Height (GH) from the TD values
- General Height (GH) was calculated as the 7th highest TD value, according to the definition provided by the Imaging and Perimetry Society
- Mean Total Deviation (MTD) is the arithmetic average of the TD values (Its interpretation is essentially equivalent to Mean Deviation)
- Pattern Standard Deviation (PSD) was calculated as the standard deviation of the TD values, without any correction factors
- Mean Sensitivity (MS) was calculated as the average of the 52 sensitivity values (excluding the two blind-spot locations) in the 24-2 VF
- Average localised perimetric defect was also quantified for each VF cluster by calculating cluster-wise MTD and MS, as described by Garway-Heath et al.
- Global, cluster-wise and point-wise progression of damage over time were quantified using simple linear regression on TD values or their cluster or global average. Progression was only calculated on a subset of eyes with a minimum of 4 tests spanning at least 4 months.

Was the “raw” data saved in addition to the preprocessed/cleaned/labeled data (e.g., to support unanticipated future uses)?
The raw dataset is provided in two alternative formats:

- Structured JSON file
- Long format table CSV file

Is the software used to preprocess/clean/label the instances available?
No

Uses

Has the dataset been used for any tasks already?
No.

Is there a repository that links to any or all papers or systems that use the dataset?
No.

What (other) tasks could the dataset be used for?
This dataset could be used to further explore localized visual field rates and interactions of neighboring visual field locations. The size of this data set opens avenues for possible machine learning applications.

Is there anything about the composition of the dataset or the way it was collected and preprocessed/cleaned/labeled that might impact future uses?
No

Are there tasks for which the dataset should not be used?
No.

Distribution

Will the dataset be distributed to third parties outside of the entity (e.g., company, institution, organization) on behalf of which the dataset was created?
The dataset is publicly available.

How will the dataset be distributed (e.g., tarball on website, API, GitHub)?
Github (https://github.com/uw-biomedical-ml/uwhvf)

When will the dataset be distributed?
At the time of submission of the dataset paper

Will the dataset be distributed under a copyright or other intellectual property (IP) license, and/or under applicable terms of use (ToU)?
The dataset will be available for open source use under the 3 clause BSD license.

Have any third parties imposed IP-based or other restrictions on the data associated with the instances?
No

Do any export controls or other regulatory restrictions apply to the dataset or to individual instances?
No

Maintenance

Who will be supporting/hosting/maintaining the dataset?
The dataset will be hosted on GitHub

How can the owner/curator/manager of the dataset be contacted (e.g., email address)?
http://comp.ophthalmology.edu

Is there an erratum?
No

Will the dataset be updated (e.g., to correct labeling errors, add new instances, delete instances)?
No

If the dataset relates to people, are there applicable limits on the retention of the data associated with the instances (e.g., were individuals in question told that their data would be retained for a fixed period of time and then deleted)?
No

Will older versions of the dataset continue to be supported/hosted/maintained?
Yes on GitHub

If others want to extend/augment/build on/contribute to the dataset, is there a mechanism for them to do so?
Yes using git
